# Perception, experience and resilience to risks: a global analysis

**DOI:** 10.1038/s41598-023-46680-1

**Published:** 2023-11-07

**Authors:** Minh Kieu, Gayani Senanayake

**Affiliations:** Department of Civil and Environmental Engineering, University of Auckland, Auckland, New Zealand

**Keywords:** Environmental social sciences, Natural hazards

## Abstract

Individual resilience is crucial amid rising global threats, yet risk perceptions and resilience worldwide remain inadequately elucidated. This research pioneers a global-scale analysis of individual risk perspectives and perceived resilience capacities. Leveraging survey data encompassing over 120 countries, we develop novel indices quantifying subjective risk perceptions, experiences, impacts, and resilience across diverse populations. Causal analysis techniques shed light on the complex dynamics shaping individual confidence in their resilience. We unveil vast disparities in risk impacts arising from differential adaptation rates. Income perception emerges as an outsized driver of resilience globally, though its influence varies contextually. Ultimately, this study challenges universal narratives of homogeneous risk experiences and perceived resilience worldwide. The globally-representative indices and causal insights provide vital evidence to inform context-specific, demographically-attuned interventions for strengthening resilience equitably. This research underscores the urgent need for inclusive policies tailored to localised risk landscapes.

## Introduction

Risks, whether originating from natural or anthropogenic causes, represent the possibility of catastrophic events that exert profound consequences on individuals, property, and the environment. In such events, the understanding of risk perception, experience and resilience becomes crucial^1^.

Risk perception encompasses a cognitive process of how humans sense and avoid harmful environmental conditions, emphasising the role of intuitive risk judgments in evaluate potential threats. Risk perception is influenced by personal beliefs and experience, which are shaped by accepted information and ideas^2^. Earlier works by Slovic^3,4^ provide a comprehensive examination of risk perception, exploring the psychological, social, and cultural factors that influence how people perceive and respond to risks. Personal experience of disasters provides more concrete information and impacts the individual perception of risks^5^. Various existing works in literature have been exploring the relationship between risk perception and experience. The Protective Action Decision Model (PADM) by Lindell & Perry^6^ is a social-psychological framework that aims to understand how individuals and communities decide to prepare for and respond to disasters and emergencies. It focuses on the cognitive processes that lead to protective actions, such as evacuation, sheltering, or other precautionary measures. The PADM has been applied and adapted in various studies to understand and promote preparedness for different types of risks, such as floods^7^ and nuclear accidents^8^. Recently, risk perception and experience have played significant roles in shaping behaviours during the COVID-19 pandemic. For example, a survey among Iranian medical students revealed a high level of COVID-19-related knowledge and preventive behaviours associated with a moderate perception of the risks from the pandemic^9^. In Bolivia, social media exposure was found to influence the adoption of preventive attitudes and behaviours by shaping risk perception^10^. Furthermore, research in Italy confirmed the empirical distinction between affective and deliberate processes in risk perception, highlighting the complex factors that contribute to individuals’ responses to the pandemic^11^. These findings underscore the importance of risk communication and the multifaceted nature of risk perception in guiding public responses to global health crises like COVID-19.

It is clear that risk perception and experience influence the individual decision-making processes, especially in terms of preparedness and recovery from disasters^12^. The ability of individuals to respond effectively, recover and learn from unexpected dangers and disturbances is often referred to as ‘risk resilience’^13,14^. Interested readers may refer to^15^ for a reviews of state-of-the-art definitions and perspectives on disaster resilience. Resilience levels are not static but can be enhanced through situation-specific guidance, access to resources, and early intervention. Inadequate crisis responses or poor stressor management can lead to unfavorable mental health outcomes, highlighting the importance of cultivating resilience. Cultivating resilience is vital when considering that inadequate responses during crises or poor management of long-term stressors can result in unfavourable mental health outcomes^16^.

To this end, there have been recent interests in measuring resilience of individuals, within the context of both man-made and natural hazards. First and Houston^17^ examined the mental health impacts of successive disasters, emphasising the protective role of individual resilience in the context of a tornado and the COVID-19 pandemic. They advocated for a multi-system disaster resilience framework. In a different geographical context, Sammonds et al.^18^ discussed Bangladesh’s journey towards resilience, highlighting achievements and challenges in the face of climate change and disaster displacement. Zander et al.^19^ assessed community resilience across Australia, identifying correlations between perceived resilience, age, self-efficacy, and well-being. These studies collectively contribute to an understanding of resilience measurement, offering insights into various domains and methodologies. One of the most comprehensive studies is Schipper & Langston^20^, which provides an overview of resilience measurement frameworks, analysing various indicators and approaches. These studies offer valuable insights into the theory and practice of measuring resilience, highlighting key challenges and suggesting ways to improve understanding.

The measurement of resilience is related to another growing interest in understanding the factors that shape risk perception and resilience of individuals. Studies by^17,18^, and^19^ have emphasised the importance of community and individual resilience in the face of natural disasters and global pandemics. A trend towards examining the role of social connections and stigma in shaping risk perceptions is evident in the work of^21^. Meanwhile, the need for robust local evidence to enhance resilience to climate risks is highlighted by^22^. The literature also explores unique perspectives, primarily in the context of climate change^23–25^, severe weather phenomena, and the COVID-19 pandemic^1,26,26,27^. Strengths of this body of work include a diverse range of methodologies and interdisciplinary approaches.

The existing body of research on personal risk perception and resilience has largely been confined to specific geographical locales and risks, leading to two critical gaps in the literature. First, there is a noticeable deficit in studies that undertake a global-scale analysis across multiple regions and multiple major personal risks to individuals. This lack of a global perspective hampers the potential to inform future policy development aimed at bolstering personal resilience across a broad spectrum of threats to quality of life. Second, the literature is marked by a lack of causal analysis of the perception and resilience to these risks. While various methodologies have been employed, the integration of causal analysis with advanced techniques such as Bayesian Networks or machine learning remains absent. This underscores the potential for innovative approaches to unravel the complex interplay between personal perception and resilience of risks.

The current study aims to address these gaps by offering a global-scale analysis and the use of causal analysis, leveraging state-of-the-art computational techniques. This endeavour contributes to both the theoretical understanding of risk perception and resilience and offers practical implications for risk management and policy formulation. By making use of the personal risk survey data from the Lloyd’s Register Foundation World Risk Poll^28^, the scientific contributions of this paper are twofold:

- We introduce four new indices - Risk Perception, Experience, Impact, and Resilience - to provide a visual global comparison of individuals’ concerns, exposures, and preparedness towards 7 major personal harms and disasters across the globe,
- We introduce a causal discovery and inference analysis to shed lights into the underlying dynamics that shape resilience at a global level, while underscoring the pivotal role of individual experiences and perceptions in this context.

The remainder of this paper includes an introduction to the dataset (Section 2), a Methodology section where we introduce the new indices and the causal analysis methods (Section 3), a Results and Discussion (Section 4), and finally the conclusion of the study.

## Dataset

The Lloyd’s Register Foundation 2021 World Risk Poll dataset is a comprehensive global survey on individuals’ risk perception, experience, and resilience across a wide range of potential threats^28^. One of the largest surveys of its kind, the dataset includes responses from over 125,000 people in 121 nations and territories. The poll covers a wide range of risk-related subjects, such as traffic crashes, natural catastrophes, health crises, food and water safety, community violence, and digital security, among others. Demographic information such as age, gender, education, and socio-economic status are also included to enable in-depth analysis of how these factors influence risk perception and resilience.

Approximately 1,000 individuals aged 15 and older from each nation were surveyed using either telephone or in-person interviews, however, in countries with larger populations such as China, Russia, and India, larger samples were employed. There may exist a response bias among those who do not partake. Additionally, people’s understanding of risk perceptions or experiences is inherently subjective and can be shaped by factors such as language and cultural influences.

From the original World Risk Poll dataset of 208 variables, we remove 139 variables that are deemed as not suitable for the analysis, such as the specific region in 121 countries, the variables from the 2019 version of the survey, the country ID, weight and a few variables that are used to form another variable within the same dataset. The remaining 69 variables are used for the proposed methodology. A full list of variables used are included in the Appendix A.

## Methodology

This paper proposes a comprehensive framework to capture the multifaceted dimensions of individual risk perspectives and perceived capacities globally. The subjective nature of risk perceptions, coupled with varying exposures and adaptive abilities across different contexts, necessitates a robust methodology integrating perceived risks and resilience metrics across diverse populations.

### Quantifying perception, experience, impact and resilience by global indices

A salient contribution of the study is the paired quantification of the self-reported experience and subjective perception of risks. We first define a ‘Perception index’ as a holistic measurement of how individuals perceive risks and harms. The Perception index $p_{i,c}^k$ of individual *i* in country *c* is defined per individual risk *k*, with the values ranging from 0 (for “not worried”) to 0.5 (for “somewhat worried”) and to 1 (for “very worried”). The Perception index for each country is the mean value of perception score from each respondent in that country.

1 $$\begin{aligned} P^k_c = \frac{\sum {p_{i,c}^k}}{N_c} \end{aligned}$$

Here $P^k_c$ is the perception index for risk *k* (e.g. crime) for country *c* (e.g. New Zealand). $N_c$ is the total number of respondent from country *c*. The larger the index, the more worry a person from the country would be for a certain risk. We examine all risks that the World Risk Poll surveyed: crime, weather, traffic accidents, food safety, water access, mental health, and workplace accidents. These personal risks both align with major frameworks of risk perception research, but also enables our Perception index to be broader than most studies in literature, as most focuses on specific risks^29^. As outlined in foundational work by Slovic^3^ on risk perception, factors directly threatening health and safety typically elicit high levels of public concern. Our inclusion of risks spanning security, infrastructure, health, and occupational hazards captures this spectrum of fundamental individual risks identified as priorities globally. The World Risk Poll, the dataset we are using, is itself grounded in seminal theoretical frameworks including the Psychometric Paradigm and Cultural Theory of Risk^28^. Thus, the selection of risks builds upon established risk perception knowledge.

However, there are always a gap between what people are worry about with what they actually experienced. As emphasised by seminal works like Lindell and Perry’s Protective Action Decision Model^6^, direct experience with a risk fundamentally shapes risk perceptions and behaviours. The survey also asked whether a person personally experienced, or knows someone who has experienced harm in the last 2 years. This enables us to propose the Experience index for each risk, offering a concrete metric of tangible harm encounters and aligning with the empiricist view that measurable phenomena constitute risk realities^4,5^. This aligns with research utilising experience-based metrics of disaster impacts^30^, asset losses, or household effects^31^ across hazards like floods, contamination, and storms.

2 $$\begin{aligned} E^k_c = \frac{\sum {e_{i,c}^k}}{N_c} \end{aligned}$$

Here $E^k_c$ is the harm experience index for risk *k* for country *c*. $e_{i,c}^k$ is the individual *i*’s from country *c* experience of risk *k*, and $N_c$ is the total number of respondent from country *c*.

While the Perception index captures individuals’ assessment of the magnitude or severity of a risk, the Experience index measures the frequency or intensity of their self-reported encounters with the hazard. This leads us to the establishments of an ‘Impact index’ as the product of Perception and Experience indices. The rationale behind this approach lies in the fact that both perception and experience play crucial roles in determining the overall effect of a risk. The notion of multiplicatively combining experience and perception has roots in Dual Processing Theories^32^ which posit both implicit and explicit factors shape reactions. By multiplying these indices, we create a composite measure that accounts for the combined influence of perception and experience on the overall impact to an individual. This multiplicative interaction implies that the impact will be more pronounced when both perception and experience are high (both has a range of 0 to 1 for each individual), highlighting the exacerbating effect of frequent or intense experiences on the perceived risk.

3 $$\begin{aligned} i^k_{i,c} = p^k_{i,c}\times e^k_{i,c} \end{aligned}$$

Where $i^k_{i,c}$ is the individual impact index for risk *k* for individual *i* from country *c*. $p^k_{i,c}$ and $e^k_{i,c}$ are the individual perception index and experience index for risk *k* for country *c*, respectively. Larger $i^k_{i,c}$ indicates a higher impact to a certain risk *k*. Then similarly, the country-wide Impact index for a particular risk *k* can be estimated by averaging the individual impact index from each individual *i*

4 $$\begin{aligned} I^k_c = \frac{\sum {i_{i,c}^k}}{N_c} \end{aligned}$$

Leveraging the knowledge from the previous section, we establish a new individual resilience index of major risks from the World Risk Poll survey dataset. Resilience refers to the capacities of individuals and systems to withstand, adapt, and recover from adversities. It encompasses both “bouncing back” to regain prior functionality after a disruption, as well as “bouncing forward” to adaptively change to better withstand future events. Individual and community resilience to major risks and natural disasters thus involves capacities to cope with challenging situations in the moment, coupled with longer-term abilities to bounce back at minimum or ideally bounce forward to enhanced functionality during the recovery process. A country-wide resilience index could then be defined as follows:

5 $$\begin{aligned} R_c = \frac{\sum {\sum {r_{i,c}^m}}}{N_c \times N_m} \end{aligned}$$

Where $r_{i,c}^m$ the resilience score of an individual *i* from country *c* on a particular question *m*, $N_c$ is the total number of people from country *c*, and $N_m$ is the total number of resilient-related questions.

The proposed Resilience index provides a subjective measure of perceived resilience capacities at the national level, quantified based on survey responses to eight survey questions cover key aspects of resilience capacity including disaster preparedness, resource adequacy, household support, and confidence in institutional readiness. As highlighted in review papers on resilience measurement like Schipper and Langston (2015)^20^, these subjective capability assessments represent important perceptual dimensions of resilience at the individual and community level. The questions map onto common resilience themes around anticipation, coping, adaptation, and recovery. Finally, the resilience factors analysed resemble those incorporated into resilience measurement frameworks like the Conjoint Community Resiliency Assessment Measure^33^, thus align with accepted resilience measurement approaches. While limited in scope, this measurement of subjective resilience perceptions offers insights to complement the objective risk metrics encapsulated in the Experience and Impact indices.

### Causal discovery analysis using Bayesian networks

The proposed indices address the need for a holistic and globally representative evaluation. The Perception and Experience indices incorporate both subjective and objective risk measures respectively, while the Impact index integrates them to gauge overall effects. Finally, the Resilience index evaluates capacities to withstand and recover from adversities. However, in the context of a globally interconnected and dynamically evolving environment, the relationships between individual concerns, exposures, and preparedness for personal risks are not simply correlative but inherently causal in nature. For policymakers, researchers, and practitioners working on global risk perception and resilience, understanding these causal pathways is paramount. It facilitates the design of interventions that target the root causes rather than the symptoms, enhancing their efficacy and efficiency. In a domain as intricate and consequential as resilience, where multi-layered factors interplay, identifying causal relationships is vital for nuanced insights that can guide strategic decisions.

Traditional statistical methodologies that prioritise correlation can be insufficient and, at times, misleading, as they are prone to the conundrum of spurious correlation, where variables may appear to be related but are influenced by unseen confounding factors. The mantra “correlation does not imply causation” epitomises this dilemma, where observational correlations may fail to reveal the underlying causal structures that govern the phenomena in question. Causal analysis transcends this limitation by systematically investigating the directional relationships between variables, discerning not only the existence but also the nature of their interconnections. In doing so, causal analysis not only detects the mere association between factors but explicates the mechanism through which one variable influences another.

The first step in causal analysis is to identify the underlying causal relationships among variables within a system, often through the use of statistical and machine learning techniques to discern the directional influences that one variable exerts on another. We adopt a Bayesian Network (BN) for this task of mapping the conditional dependencies and causal structures linking key variables.

The probabilistic graphical models underlying Bayesian Networks excel at representing the conditional independence relationships between multiple interacting variables within intricate systems. By encoding variables as nodes and their conditional dependencies as edges, Bayesian Networks can map the potential causal structures and pathways underlying a phenomenon. A BN is represented as a directed acyclic graph (DAG) where each node corresponds to a variable and is accompanied by a conditional probability. The latter is contingent upon the variables that are situated upstream within the DAG structure. Often the process of developing an BN consists of two primary phases: (1) learning the structure and (2) estimating the parameters. The process of causal discovery within the Bayesian framework is primarily concerned with the first phase of BN modelling, which is learning the structure or the graphical representation.

The learned graph from this phase often represents a causal structure, where the nodes represent variables and the edges represent causal relationships. This graphical causal mapping avoids the common pitfall of spurious correlations that can misrepresent merely coincidental associations as causally meaningful. By modeling the entire system of conditional dependencies, Bayesian Networks identify relationships more likely to be genuinely causal versus spuriously correlative. Additionally, the inductive learning capability of Bayesian Networks enables inference of network structure from data, allowing causal discovery without relying solely on assumptions. By combining probabilistic graphical models with inductive learning, Bayesian Networks offer a rigorous yet flexible methodology for data-driven causal mapping. This approach has recently been explored in literature. Tanaka and Jones (2023) demonstrated the use of DAG to decipher the complexities of chronic liver disease epidemiology, providing a transparent way to identify and illustrate causal relationships between variables^34^. Similarly, Wei et al. (2022) employed DAG in the structure learning of protein signaling networks, considering causal directions between variables as constraints, thereby improving the structure of the Bayesian network^35^. Sobieraj and Metelski (2022) applied DAG in the analysis of the British housing market, using Bayesian network analysis to find the most appropriate relationships between variables^36^. In the field of healthcare, a study on hospital-acquired pediatric venous thromboembolism utilized DAG to identify confounding and multiple causalities, clarifying the influence of various risk factors^37^. These studies collectively highlight the versatility and efficacy of DAG in representing and understanding causal relationships across different fields.

During this shape learning stage, the dependency graph’s structure is deduced from the data. Simple scenarios involving fewer variables are amenable to an exhaustive exploration of potential dependency graph structures. These structures can be evaluated using criteria such as the Bayes Information Criterion. Nonetheless, the growing complexity of DAGs based on the number of labelled vertices makes exhaustive search computationally unfeasible beyond a threshold of approximately four to five variables, necessitating more sophisticated search techniques. One such method, the Hill Climbing Algorithm, operates as a greedy algorithm initiating with an isolated network hypothesis. It subsequently integrates individual arcs based on the augmentation of the scoring criterion. While efficient in its execution and resource utilisation, this algorithm exhibits a major drawback of converging upon encountering a local minimum. Despite this limitation, the algorithm’s speed and satisfactory results offer a compelling case for its usage in this paper.

### Causal inference using causal forest double machine learning (CF-DML)

A DAG obtained from causal discovery often only represents the skeleton of the causal structure - it shows which variables are directly and indirectly connected, but the direction of the arrows might not be accurate, without additional assumptions or domain knowledge. Causal discovery also does not quantify the strength of these causal relationships, or the causal effects.

Given the potential value of an index (Perception, Experience, Impact or Resilience), we aim to measure the causal impact of an specific ‘treatment’ variable, e.g. how much a personal experience with a crime cause a change in individual Impact index? Mathematically:

6 $$\begin{aligned} \tau _i = Y_i(1) - Y_i(0) \end{aligned}$$

Where $\tau _i$ is the individual treatment effect of person *i*, $Y_i(1)$ and $Y_i(0)$ are two different outcome given two different values of treatment.

7 $$\begin{aligned} Y^{obs}_i(t)= {\left\{ \begin{array}{ll} Y_i(1), &{} \text {if } treatment = 1\\ Y_i(0), &{} \text {if } treatment = 0 \end{array}\right. } \end{aligned}$$

If the treatment variable is continuous, we will then have $\tau _i = \partial Y(t)$. In an ideal world, the development of the dataset should have gone through rigorous randomised controlled trials, where individuals are randomly assigned to treatment or no-treatment. However, real-world datasets (such as our World Risk Poll dataset) are often not randomised controlled, thus the real data often only has one of treatment and one outcomes and the actual individual treatment effect is always unobservable. This leads us to the fundamental concept of average treatment effect (ATE). It quantifies the expected (or average) difference in outcomes if all units in the population were to receive the treatment versus if none of them were to receive the treatment.

8 $$\begin{aligned} \tau = E[Y_i(1) - Y_i(0)] = E[\tau _i] \end{aligned}$$

Various methods, such as regression and matching methods, can be used to estimate the ATE by creating comparable treatment and control groups. While ATE provides a powerful measure of the overall effectiveness of a treatment or intervention across a population. It gives us a single number representing the overall effectiveness of the treatment and overlooks important heterogeneity in treatment effects across different countries, regions or individuals. This leads us to the concept of the Conditional Average Treatment Effect (CATE), also known as treatment effect heterogeneity. CATE is defined as the expected difference in outcomes between treated and untreated units, given a set of covariates or characteristics (X):

9 $$\begin{aligned} \tau (x) = E[Y_i(T)-Y_i(0)|X] = E[\tau _i|X] \end{aligned}$$

Here, $\tau (x)$ is a vector of CATEs corresponding to the different treatments in $T$, a multi-dimensional treatment vector. $Y_i(T)$ represents the potential outcomes under the multi-dimensional treatment vector $T$, and $Y_i(0)$ represents the potential outcomes under the control condition. $X$ are the set of observed covariates. We focus on estimating CATE instead of ATE because the initial data analysis suggests that there are noticeable heterogeneity across different countries and age group. For example, the income perception of a person from a wealthy country might be significantly different to a undeveloped country. By estimating the CATE, we provide more personalised policy implications to improve personal resilience.

To achieve this, we turn to Causal Forest Double Machine Learning (CF-DML), a state-of-the-art method based on Generalised Random Forest^38^ to furnish unbiased causal effect estimates. At its core, CF-DML employs a two-step process that separates the task of controlling for confounding from that of estimating causal effects, thereby mitigating the bias usually encountered in traditional methods. Utilising Random Forest algorithms for both the treatment and outcome models, it captures complex and non-linear relationships in the data, allowing for robust and reliable estimates. The method is particularly well-suited for multiple treatment scenarios such as ours. Its ability to provide consistent and asymptotically normal estimates makes it an invaluable tool for policy evaluation and decision-making in complex systems.

## Results and discussions

**Figure 1:**
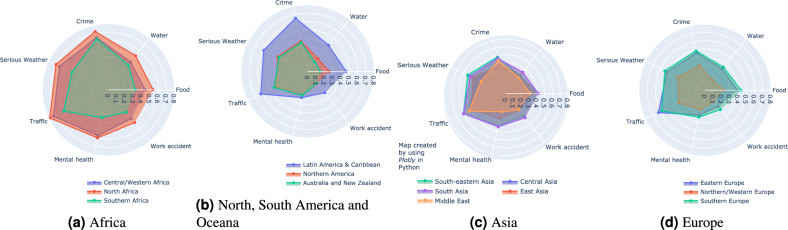
Perception index of different risks in different regions.

We first provide a global view on risk perception and resilience on Figure 1, where the mean perception index $P^k_c$ from each region is illustrated. We analyse all the risks surveyed in the dataset, namely crime, water, food, work accident, mental health, traffic crashes and serious weather events. For each risk, we give a value of 1 if the respondent is “very worried”, 0.5 if they are “somewhat worried” and 0 if they are “not worried” towards the risk. Figure 1 was created by using the Plotly Open Source Graphing Library in Python^39^, version v5.9.0.

Larger charts represent higher perception indices, indicating greater levels of worry or concern towards each respective risk. Across all regions, “crime” and “traffic” (i.e. road crashes) consistently emerge as top concerns based on the Perception index values. This indicates these two risks are viewed with high levels of worry globally. “Serious weather” and “food” risks also rank among the top concerns globally, though slightly lower than crime and traffic risks. Still, the high perception index values reveal these are seen as major risks worldwide.

Overall, people from regions with more developing nations (Africa, Latin America, Caribbean and Asia) tend to have greater levels of concern about the risks. In contrast, regions with more developed countries (Northern America, Northern/Western Europe, Australia/New Zealand) exhibit lower perception index values for the risks, indicating relatively lower levels of concern. The stark contrast between perception index values in developed versus developing regions is especially noticeable for traffic, crime, serious weather, and food risks. The pattern suggests individual risk perceptions are shaped by the level of economic and social development of one’s geographical region.

**Figure 2:**
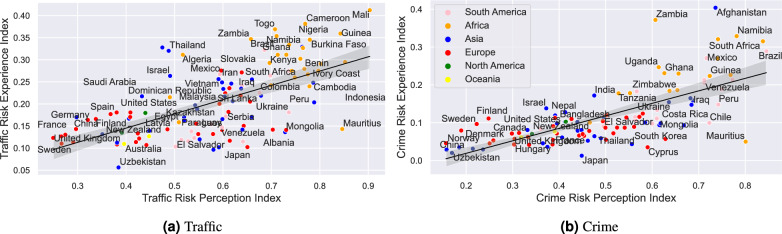
Risk perception index vs experience index of Traffic and Crime.

Figure 2 shows a comparison between how people perceived risks ($P^k_c$) and their self-reported experience ($E^k_c$). Here the value of $e_{i,c}^k$ is either 0 (no harm), 0.25 (know someone who experienced harm), 0.5 (experienced harm) or 1 (both).

We only show Traffic and Crime risks on Figure 2, the remaining 5 risks can be found in Appendix B. Countries perform better if they are close to the bottom left corner of the graphs (both Perception and Experience indices are low) rather than being in the top right corner. Figure 2 shows that there is a clear positive correlation between the risk perception index and experience index, indicating that actual experience of harms is associated with greater perceived risk levels. This aligns with the existing empirical data in literature showing that direct exposure or experience with a risk can heighten subjective perceptions of that risk^5,11^. However, the strength of correlation varies - it is stronger for traffic risk than crime risk, suggesting additional factors also influence crime risk perceptions. Many European and Asian countries, especially developed nations, cluster toward the bottom left corner, reflecting both lower risk perception and experience indices, whereas African nations are predominantly concentrated in the top right corner. Countries which are located below the regression line are generally concerning more about risks than they actually experienced when in comparison to other countries. We find more European and Asian countries on this side, whereas the top of the regression line has more developing countries, especially those in Africa.

Finally, a new Resilience Index is proposed using 8 questions related to personal resilience in the World Risk Poll dataset:

- Suppose you lost all of your household income, how long would you be able to cover your basic needs?
- Do your neighbours care about you and your well-being?
- Is your national government well prepared to deal with a disaster?
- Are your hospitals well prepared to deal with a disaster?
- Are you and your family well prepared to deal with a disaster?
- Is your local government well prepared to deal with a disaster?
- Could you protect yourself and your family in a future disasters?
- Is the plan for future disaster known by all household member in your family?

Similar to the way of defining other indices, we encode the answers for these questions with a value between 0 to 1. We assign 1 to the most resilient answer (e.g. “Yes, well prepared”), 0 to the least (e.g. “No, not well prepared”) and 0.5 to the unsure answer (e.g. “It depends”). Since the answers are subjective, the new Resilience index measures perceived resilience, or how much an individual thinks they can recover and adapt to a disaster. Here “disaster” refers to major adversity events in general. They can arise from both natural and anthropogenic causes, e.g. financial security or failure of vital infrastructure.

**Figure 3:**
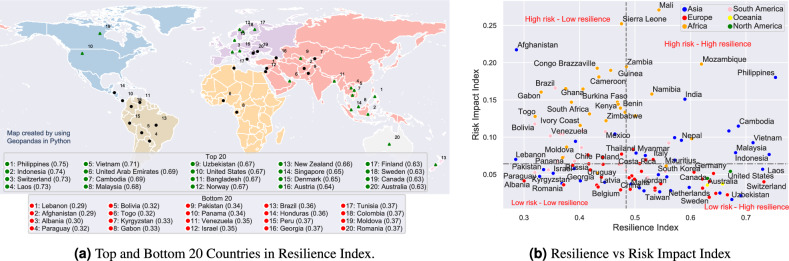
Resilience Index.

Figure 3(a) shows the top and bottom 20 countries in Resilience Index and reveals distinct patterns across the globe. Here the map was created by using Geopandas 0.13.2 in Python^40^. In the top 20, the presence of Southeast Asian countries such as the Philippines, Indonesia, Laos, Vietnam, and Cambodia is remarkable. These countries, despite being frequently exposed to natural disasters due to their geographical location, have residents who believes in themselves, their communities and local governments (high perceived resilience score). This is possibly driven by effective local community engagement, robust disaster preparedness strategies, and tailored infrastructure adaptations^41^.

The high Resilience index scores in economically prosperous countries like the United Arab Emirates, Switzerland, United States, and Singapore show that their citizens believe in the substantial investments in infrastructure, technology^42^, and public health,^43,44^ coupled with strong governance and institutional frameworks that enable effective risk management and disaster response. Among the Nordic countries - Norway, Denmark, Finland, and Sweden - renowned for their high-quality public services, community trust, and comprehensive social security systems, their high Resilience scores underscore the importance of social factors and public services in promoting societal resilience^45^.

On the other hand, the bottom 20 countries present a contrasting scenario. Lebanon and Afghanistan, both grappling with prolonged political instability, insecurity, and economic crises, exhibit the lowest Resilience scores. This suggests that political stability and security are key determinants of perceived resilience. The presence of Eastern European and Latin America countries such as Albania, Paraguay, Bolivia, and Moldova indicates that economic challenges, including poverty and income inequality, might negatively impact subjective resilience among residents^46^. This could be due to limited resources for investment in risk mitigation and disaster recovery, along with weaker public institutions and infrastructure^47,48^.

Moreover, it’s worth noting that some countries, such as Israel and Brazil, which have advanced technological capabilities or significant economic size, also fall into the bottom 20. This might hint at the complex nature of resilience, where socioeconomic disparities, internal conflicts, or uneven distribution of resources could detrimentally affect a nation’s resilience despite its overall technological or economic prowess^49^.

Figure 3(b) compares the proposed Resilience index with the Risk Impact index. Here the two dashed lines show the median values for the two indices. Median has been chosen instead of mean to ensure a similar number of countries in each category defined by the two median lines. Generally, there is not much correlation between these two indices as a country could belong to any of the 4 categories. Figure 3 shows the values of Risk Impact and Resilience Index. We name the 4 resilience categories as follows:

**High risk - Low resilience.**: This category comprises countries that bear substantial exposure to personal harms and disasters, coupled with a lack of perceived resilience to these risks. Predominantly, many Latin America and African nations fall into this category, though there are notable exceptions for countries which current are or has recently been in humanitarian crises such as Afghanistan.
**Low risk - Low resilience.**: Countries in this group are characterised by their limited exposure to harm and disaster, yet they similarly lack of subjective resilience to risks. This category exhibits a diverse representation, with countries from various regions except North America and Oceania, including Russia, China, Poland, Pakistan, and Romania.
**Low risk - High resilience.**: Nations in this category are both minimally exposed to risks and exhibit high resilience to potential disasters. This is arguably the most favourable state for any country and should serve as a benchmark for others. Notably, many developed nations such as Germany, Sweden, New Zealand, Canada, South Korea, and Switzerland belong to this group, along with certain highly resilient developing countries like Indonesia, Laos, and Uzbekistan.
**High risk - High resilience.**: This final category includes countries that, while experiencing high exposure to harm and disasters, demonstrate significant adaptability. Respondents from these countries report a high impacts of hazards to their lives, yet possess a strong belief in their abilities to adapt to adversaries. Countries that are traditionally associated with frequent risks and disasters, such as India, Vietnam, Nepal, the Philippines, and Mozambique, fall under this classification. However, given their dense populations and high Risk Impact indices, tailored policies are necessary to transition them toward the Low risk - High resilience category.

**Figure 4:**
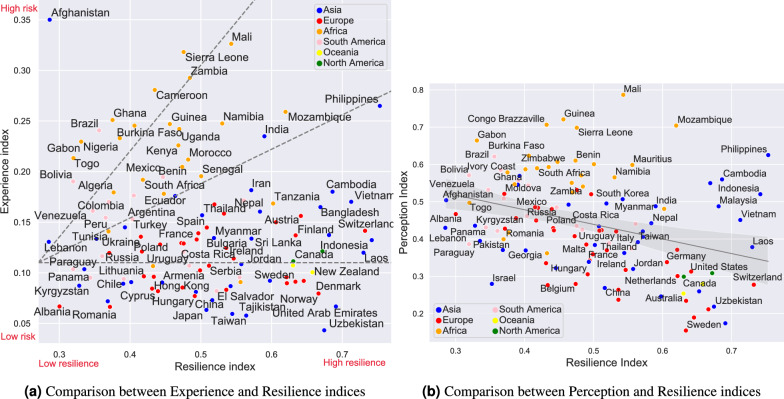
Resilience vs Perception index and Experience index.

We investigate the interplay between the Experience and Resilience indices that we propose, as depicted in Figure 4(a). To elucidate the relationship, we overlay three dashed lines representing distinct patterns observed in the data.

Two of these patterns, represented by the diagonal lines, signify that an increase in exposure to harms and disasters typically correlates with a heightened state of perceived resilience. Yet, the rate of this adaptation varies significantly from one country to another. Specifically, countries located along the upper diagonal line, such as Mali, Sierra Leone, Zambia, and Cameroon, exhibit a relatively slower belief in their adaptation to harms. This observation is consistent with their designation as High risk - Low resilience, as categorised in Figure 3(b). This implies that the primary reason for these countries falling into the High risk - Low resilience category could be their comparatively slower rate of adaptation to the risks they face.

In contrast, countries located along the lower diagonal line, including the Philippines, Mozambique, and India, appear to adapt more swiftly to harms, thereby showcasing a stronger belief in their abilities to bounce back and bounce forward from disasters. Many of these countries fall into the High risk - High resilience category, further substantiating the observation that a faster adaptation rate to risks leads to a stronger perceived resilience, even in the face of high risk exposure.

Lastly, the horizontal dashed line indicates a group of countries with consistently low exposure to harms and disasters, as evidenced by their low Experience Index. Interestingly, this group exhibits a dichotomy in terms of perceived resilience. People in some of these nations reported a lower resilience to disasters, falling into the Low risk - Low resilience category, while others reported a high resilience, thereby belonging to the Low risk - High resilience category. This divergence emphasises that low exposure to risk does not directly translate into high belief in resilience, further underscoring the multifaceted nature of risk resilience.

Figure 4(b) suggests a subtle, yet discernible, linear correlation between Perception and Resilience indices. Specifically, countries demonstrating a higher degree of perceived resilience tend to exhibit lesser concern regarding harms and disasters, relative to their less resilient counterparts. This aligns well with several studies in literature, such as in Eady et al. (2020), where respondents who are confident in their resilience exhibited lower levels of risk concern about heat waves^21^. A closer scrutiny of the figure reveals that countries situated below the regression line generally display a level of perceived resilience that surpasses the anticipated perception of risks when we consider the global context. This subset of nations includes many developed countries from Europe, Asia, Northern America, and Oceania, underlining the strong performance of these regions in terms of risk resilience. Conversely, the countries located above the regression line can be divided into two major cohorts: the less resilient African countries, and the highly resilient Asian countries, predominantly from Southeast Asia. The African cluster comprises countries like Guinea, Sierra Leone, Benin, and Zimbabwe, while the Asian cohort includes nations such as the Philippines, Cambodia, Indonesia, and Malaysia. Notwithstanding the palpable resilience gap separating these two groups, it is intriguing to observe a shared attribute - a high level of risk concern. Thus while a strong correlation between Perception and Resilience indices can be found, similar to Eady et al. (2020)^21^, this unifying trait underscores a noteworthy pattern: the level of worry about risks is not exclusively tied to a nation’s confident in resilience. Instead, it can persist irrespective of the country’s capacity to withstand harms and disasters, emphasising the complex interplay between perceived risks and resilience capabilities.

### Causal discovery results

**Figure 5:**
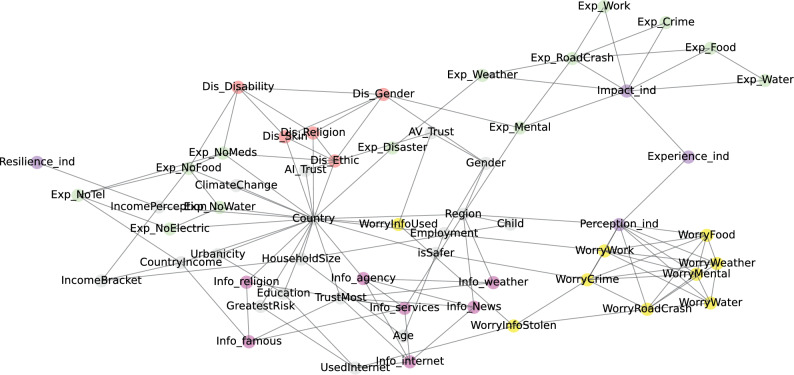
Bayesian Causal network of the World Risk Poll data.

Figure 5 illustrates the final causal network structure resulted from the causal discovery analysis using Bayesian Network. Each node in the graph corresponds to a variable, and each directed edge between nodes represents a conditional probability distribution.

Figure 5 provides a holistic visualisation of the conditional dependencies underlying the proposed indices and variables constituting individual risk perspectives and perceived resilience worldwide. Several key features stand out from the figure.

Firstly, fundamental demographic variables like age, gender, education level, and country of residence occupy pivotal positions near the top of the network, underscoring their overarching influence on downstream factors. The network also incorporates various mentalities and social dynamics variables around *Country*, such as measures of discrimination, trust in institutions, experience of major shortage of commonalities and feelings of safety to map their roles in orienting risk interpretations. This makes intuitive sense as all other variables seem to be dependent on where the respondent is from, and their life experience.

Secondly, variables capturing household characteristics, access to key resources and infrastructure, and information sources showcase the importance of socioeconomic environment in impacting risk-related attitudes and behaviours. The group of variables that telling where individuals are seeking for information ($Info\_$ variables, located at the far left side of the figure) decides where individual will trust most (the *TrustMost* variable). Interestingly, if a respondent looks for information from the Internet ($Info\_internet$), we will know that they do use it (*UsedInternet*), and that they are likely to worry about their information being stolen or used (*WorryInfoStolen* and *WorryInfoUsed*).

Thirdly, the network highlights the interconnected nature of subjective risk views (through the Perception index and its related variables) and objective exposures (through the Experience and Impact index). The Perception index integrates numerous risk perception variables, representing subjective views regarding threats like crime, weather, traffic, and water access. Meanwhile, the Impact index assimilates variables capturing objective harm encounters across those risk domains. This confirms the validity of the causal network, because the Perception and Impact indices have been defined using these variables. The multiplicative linkage from Perception and Experience to the Impact index highlights the Experience index’s role in encapsulating the interplay between subjective risk interpretations and tangible exposures, and underscores the bidirectional relationship between subjective risk views and objective harm.

Finally, the position of the Resilience node as the ultimate child in the network emphasises its nature as a multifaceted emergent property, arising through complex pathways from foundational demographics to risk experiences and attitudes. an integrative outcome, shaped by the complex interplay of demographics, infrastructure, experiences, attitudes, and social environments. Despite the diversity of variables in the network, the Resilience index has only one direct parent, the *IncomePerception* node. This solitary direct connection underscores income perception as a pivotal precursor exerting a sizeable causal influence on individual’s confidence in their resilience globally.

### Causal inference analysis

The DAG in Figure 5 shows interesting causal relationship between the four proposed indices with other variables in the World Risk Poll dataset. This section aims to confirm and quantify these causal relationships by using machine learning techniques to estimate the causal effect through Conditional Average Treatment Effect (CATE). We adopt Causal Forest Double Machine Learning (CF-DML)^38^ to estimate CATE, because the method allows us to control for a plethora of observed confounders and also efficiently handles multiple treatments, offering a comprehensive landscape of the treatment effects across different population group.

**Figure 6:**
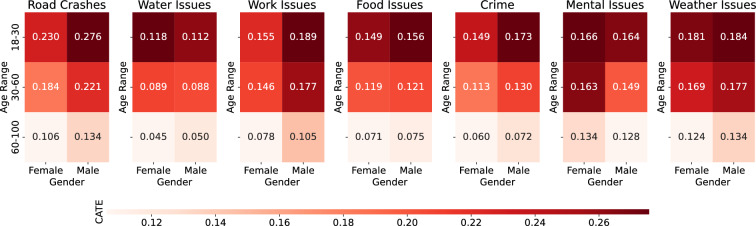
Causal effect of risks to the Impact index in different Age and Gender.

We first aim to quantify the causal impact of individual experience to risks to the proposed Impact index. Figure 6 shows the heterogeneity of Age and Gender when looking at the causal impact of each of the 7 experienced risks. Since the experienced risks cause the Impact index (see Figure 5), we visualise the value of CATE for the Impact Index only. The outcome variable *Y*, represented by $Impact_ind$, quantifies an “Impact Index” and is postulated to be influenced by both treatment and control variables. The treatment vector *T* comprises seven dimensions, reflecting the seven risks that we are studying in this paper, as also discussed in Figure 1. Finally, all other variables are treated as Control variables *W* for the CF-DML model.

The estimated Conditional Average Treatment Effects (CATE) across age and gender segments elucidate salient heterogeneity in the causal impacts of diverse harm experiences on individual harm impact. Darker colours and greater values denote intensified causality between the risk exposure and harm impact among particular demographic subgroups. While all CATE values are positive, reflecting universally detrimental effects across groups, certain hazards like traffic crashes and meteorological events exhibit appreciably elevated CATE relative to other threats, underscoring their disproportionate impact.

A predominant pattern is the decline in CATE magnitude with advancing age for nearly all treatment types. This implies that the causal effects of harm experiences fundamentally weaken among elderly populations, potentially indicative of progressive belief in resilience accumulation across most harm domains. This aligns with inoculation theories of lifespan development and resilience, which posit the strengthening of protective capacities through lived experience^50^. Compelling gender variations can also be seen. Females exhibit appreciably lower CATE relative to males for a majority of risks, especially older females. Regarding specific risks, substantially elevated CATE emerges among males for occupational, traffic, and crime threats.

**Figure 7:**
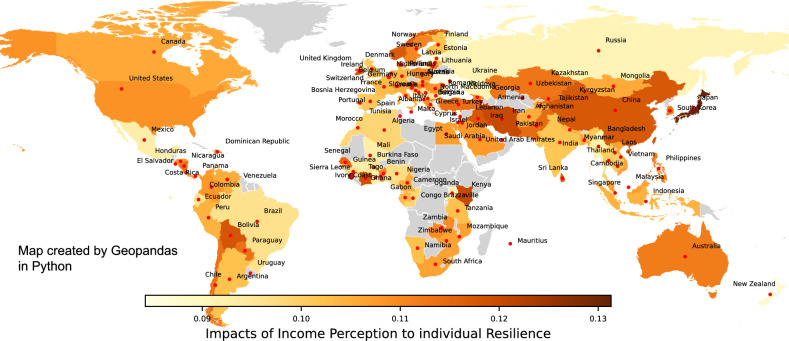
CATE of Income Perception globally.

Finally, we aim to quantify how we can make people become more resilient by improving their income, through the value of *IncomePerception*. Here CF-DML model uses $Resilience_ind$ as the outcome variable *Y*, *IncomePerception* as the sole treatment variable, and all other variables as Control variables *W*. Similar to the previous analysis, we are interested in the heterogeneity of causal impacts to subjective resilience. Figure 7 shows how Income Perception affect Resilience index in different country. The map here was again created by using Geopandas 0.13.2 in Python^40^.

Figure 7 shows that in general, a unit improvement in Income Perception (i.e. from “finding it very difficult on present income” to “living comfortably on present income”) would improve the Resilience index of an individual by up to 13%. However, the effect varies between countries, where developed nations in North America, Western Europe, and Oceania predominantly exhibit highly positive CATE, implying income perception substantive positive effects on perceived resilience amid abundant socioeconomic resources^51^. Conversely, muted CATE in African and Latin American countries despite heightened income instability highlights that financial standing alone inadequately captures lived experiences within developing contexts marked by institutional and infrastructural limitations. Meanwhile, the high variability within Asia showcases the insufficiency of regional generalisations. By spotlighting such heterogeneity, CATE provides vital insights to inform context-specific, evidence-based policies for cultivating resilience equitably worldwide.

## Limitation

Related to the concepts of risk and resilience, robustness is another interconnected idea that primarily refers to a system’s capacity to withstand pressures and prevent disruptions, particularly in response to threat impacts. Robustness is more applicable to engineered systems that maintain functionality under duress, rather than to individuals who face disruptions. While the inclusion of robustness could enrich our understanding of systemic resilience, the primary focus of the current research is on risk and resilience at the individual level. Future work may aim to integrate perspectives on both individual-level resilience and system-level robustness.

A limitation of this study is the reliance on subjective metrics of perception and experience to represent risk, which may not fully capture objective risk levels. While the proposed indices quantify self-reported risk assessments, they should be interpreted as subjective rather than absolute measures of risk realities. Additionally, comparing subjective risk metrics across different global contexts requires careful interpretation, as risk perceptions and experiences can be shaped by various contextual factors. Future work to integrate objective risk data could validate and strengthen the comparability of the proposed indices.

## Conclusion

This research elucidates the complex interplay of factors shaping individual risk perspectives and perceived resilience capacities amid diverse global populations. The proposed framework introducing 4 new subjective indices, coupled with causal analysis techniques, furnishes nuanced insights hitherto unexplored in literature.

The Perception, Experience, Impact, and Resilience indices introduced in this study integrate core aspects of leading risk and resilience frameworks while providing novel contributions. The Perception index quantifies subjective risk interpretations, aligning with decades of risk perception research. The Experience index captures self-reported disaster encounters, mirroring common usage in literature. In combining these to represent overall harm impacts, the Impact index uniquely integrates analytical and experiential processes shaping reactions. Finally, the Resilience index synthesises key perceptual factors underlying adaptive capacities. While building upon accepted risk and resilience knowledge, these indices make several advances.

First, the globally inclusive scope compiling perceptions across diverse populations advances cross-cultural understanding. Second, while the proposed indices are still perceptual measures rather than objective realities, they quantify both concrete risk exposures and abstract risk perceptions, highlighting significant disparities in risk impacts and resilience arising from differential adaptation rates across multiple risks and hazards to individuals. As noted previously, these indices represent subjective metrics based on self-reports rather than externally validated assessments. Finally, the individual-level causal analysis spotlights contextual heterogeneity in the dynamics driving resilience, underscoring that singular policies cannot cater to diverse populations.

By expanding the measurement and comparability of integral risk and resilience dimensions, this study enriches multi-hazard evaluation and informs evidence-based policies to strengthen community safety worldwide. The findings reveal particular risks and demographics warranting prioritised policy attention in strengthening resilience equitably worldwide. Specifically, the slower adaptation of certain developing nations to frequent harms underscores the urgency of investments in protective infrastructure and targeted risk communication. Meanwhile, the outsized influence of income perception on perceived resilience globally compels nuanced interventions tackling financial insecurity amidst diverse socio-cultural milieus.

Ultimately, by elucidating the multiplicity inherent in risk experiences and resilience capacities worldwide, this research challenges monolithic narratives and solutions. It argues for inclusive, evidence-based policies tailored to local contexts, demographics, and risk landscapes. Moving forward, the proposed framework and techniques could be leveraged to inform context-specific interventions that reflect the diversity of lives threatened by risks worldwide. This could pave the way toward equitable resilience.

### Supplementary Information

Supplementary Information.

## Data Availability

The World Risk Poll dataset is available for public access at https://wrp.lrfoundation.org.uk/data-resources. For detailed methodological information on the Lloyd’s Register Foundation World Risk Poll survey, including its experimental protocol, and ethical approval or consent, please refer to the Lloyd’s Register Foundation 2021 World Risk Poll report^28^.
