# Supplementary material for: Perception, experience and resilience to risks: a global analysis
Source: Sci Rep. 2023 Nov 7;13:19356. doi: 10.1038/s41598-023-46680-1 (PMC10630481; doi:10.1038/s41598-023-46680-1)
Supplement: Supplementary file 1 — Supplementary Information. [file 41598_2023_46680_MOESM1_ESM.pdf]

1 Appendix

1.1 Appendix A

Variable	Description
Country	The country in which the participant resides.
Region	The region within the country.
CountryIncome	Income category of the country as per World Bank classification.
Age	Age of the participant.
Gender	Gender of the participant.
Education	Level of education completed by the participant.
IncomePerception	Self-perception of the participant's income level.
IncomeBracket	Actual income bracket of the participant.
Employment	Employment status of the participant.
Urbanicity	Whether the respondent lives in a rural area/small town or a large city/suburb
HouseholdSize	Number of individuals in the participant's household.
Child	Whether the participant has children.
isSafer	Perception of safety compared to 5 years ago.
GreatestRisk	Participant's perception of the greatest risk they face.
ClimateChange	Whether climate change is a threat in next 20 years.
WorryFood	Level of worry about food security.
WorryWater	Level of worry about water supply.
WorryCrime	Level of worry about crime.
WorryWeather	Level of worry about extreme weather conditions.
WorryRoadCrash	Level of worry about road accidents.
WorryMental	Level of worry about mental health.
WorryWork	Level of worry about job security.
Exp_Food	Experience with food insecurity.
Exp_Water	Experience with water insecurity.
Exp_Crime	Experience with crime.
Exp_Weather	Experience with extreme weather conditions.
Exp_RoadCrash	Experience with road accidents.
Exp_Mental	Experience with mental health issues.
Exp_Work	Experience with job loss or insecurity.
UsedInternet	Whether the participant has used the internet.
WorryInfoStolen	Concern about personal information being stolen online.
WorryInfoUsed	Concern about personal information being misused online.
AV_Trust	Level of trust in autonomous vehicles.
AI_Trust	Level of trust in artificial intelligence.
Info_weather	Whether the respondent look to National Weather Service for information About Disaster.
Info_agency	Whether the respondent look to Governmental agencies for information About Disaster.
Info_News	Whether the respondent look to the news for information About Disaster.
Info_religion	Whether the respondent look to religious leaders for information About Disaster.
Info_famous	Whether the respondent look to celebrities for information About Disaster.
Info_services	Whether the respondent look to emergency services for information About Disaster
Info_internet	Whether the respondent look to the Internet for information About Disaster
TrustMost	The most trusted source of information.
Exp_Disaster	Experience with natural or man-made disasters.
Exp_NoElectric	Experience with electricity shortages.
Exp_NoWater	Experience with water shortages.
Exp_NoFood	Experience with food shortages.
Exp_NoMeds	Experience with medicine shortages.
Exp_NoTel	Experience with telecommunication outages.
Dis_Skin	Experience with skin color-based discrimination.
Dis_Religion	Experience with religious discrimination.
Dis_Ethic	Experience with ethnic discrimination.

Dis_Gender	Experience with gender-based discrimination.
Dis_Disability	Experience with disability-based discrimination.
Perception_ind	Personal perception index.
Impact_ind	Personal impact index.
Experience_ind	Experience index.
Resilience_ind	Resilience index.

Table 1. A full list of variables used for Causal discovery and inference

1.2 Appendix B

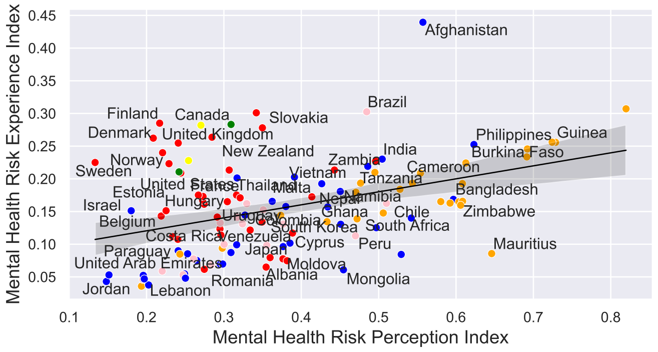

(a) Mental Health

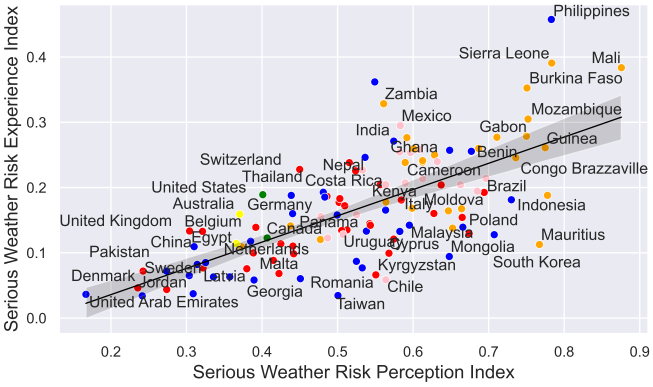

(b) Serious Weather

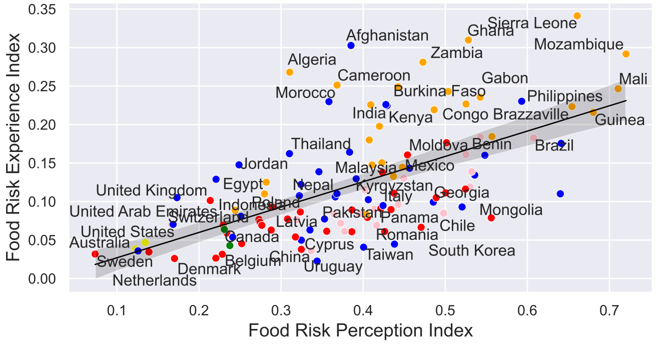

(c) Food

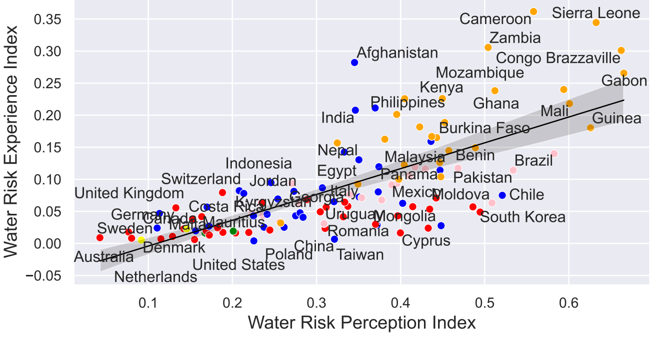

(d) Water

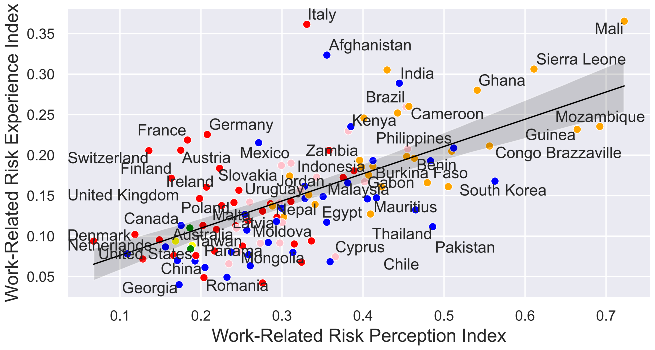

(e) Work

Figure 1. Risk perception index vs experience index of different risks in different countries

By analysing all the remaining 5 risks (apart from the traffic and crime risks on Figure **??**), we find some 'outlier countries' which need immediate action to improve both P_c^k and E_c^k, such as Cameroon, Mali in traffic, Afghanistan in crime and mental health, Philippines, Mali and Sierra Leone in serious weather events, Sierra Leone, Ghana and Afghanistan in food, Cameroon

and Sierra Leone in water, and Italy, Mali and Afghanistan in work. Remarkably, the only graphs which does not look quite non-linear are Figure 1(a) and (e), where many people in developed countries (e.g. France, Germany, Switzerland, Austria, and Finland) are facing more mental health and work-related issues compared to those in developed countries, despite the level of concerns for those issues in these countries are low.
